# Expectant Management Leading to Successful Vaginal Delivery following Intrauterine Fetal Death in a Woman with an Incarcerated Uterus

**DOI:** 10.1155/2017/2635275

**Published:** 2017-09-10

**Authors:** Masafumi Yamamoto, Mio Takami, Ryosuke Shindo, Michi Kasai, Shigeru Aoki

**Affiliations:** Perinatal Center for Maternity and Neonate, Yokohama City University Medical Center, Yokohama, Japan

## Abstract

Expectant management leads to successful vaginal delivery following intrauterine fetal death in a woman with an incarcerated uterus. Management of intrauterine fetal death in the second or third trimester of pregnancy in women with an incarcerated uterus is challenging. We report a case of successful vaginal delivery following intrauterine fetal death by expectant management in a woman with an incarcerated uterus. In cases of intrauterine fetal death in women with an incarcerated uterus, vaginal delivery may be possible if the incarceration is successfully reduced. If the reduction is impossible, expectant management can reduce uterine retroversion, thereby leading to spontaneous reduction of the incarcerated uterus. Thereafter, vaginal delivery may be possible.

## 1. Introduction

Uterine incarceration is a rare complication of pregnancy, where the enlarged retroflexed uterus becomes engaged into the small pelvis. Reported causes include pelvic adhesions resulting from a previous surgery, pelvic peritonitis, or endometriosis; uterine fibroids; and uterine malformation [1, 2]. Uterine incarceration is a rare condition, with an incidence of 1 in 3,000 to 10,000 pregnancies [3, 4].

In general, vaginal delivery is contraindicated in women with an incarcerated uterus, because this condition is associated with a high risk of intrapartum uterine rupture [5, 6]. In irreducible cases that persist close to delivery, cesarean delivery is recommended. However, in cases of intrauterine fetal death (IUFD), cesarean delivery offers no advantage; therefore, it is reasonable to attempt vaginal delivery, because cesarean delivery carries the risk of complications such as bleeding and physical and psychological burden for the pregnant woman. Thus, the management of IUFD in women with an incarcerated uterus poses a therapeutic dilemma for obstetricians.

Here, we report a case of successful vaginal delivery after IUFD by expectant management in a woman with an incarcerated uterus. We also describe a therapeutic strategy for the management of such cases.

## 2. Case Presentation

The patient was a 37-year-old primipara woman. She had a history of uterine fibroids and cystectomy due to rupture of ovarian endometrial cyst. Transvaginal ultrasound performed at 5 weeks of gestation revealed a 6 cm fibroid in the fundus of the uterus.

**Figure 1:**
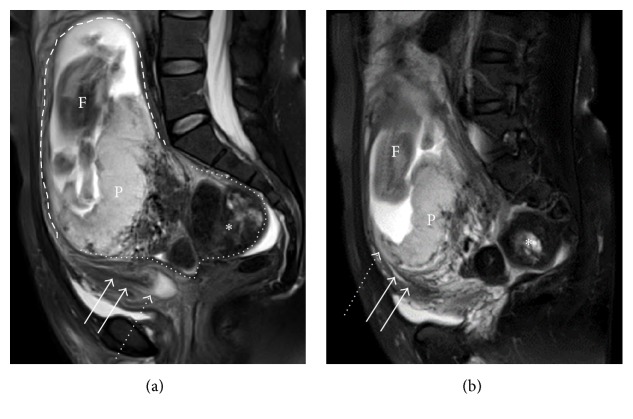
Sagittal T2-weighted magnetic resonance imaging (MRI) at 18 weeks of gestation shows a large fibroid (white asterisk) engaged in the pouch of Douglas. The cervix (white arrow) and the anterior wall of uterus (dashed line) are cranioventrally stretched. The fundus and the posterior wall of uterus (dotted line) were entrapped in the pelvis between the sacral promontory and pubic symphysis. Dashed arrows show external ostium of uterus (a) and internal one (b). F = fetus; P = placenta (a, b).

At 16 weeks and 6 days of gestation, the patient developed abdominal pain and genital bleeding and was admitted to our hospital. On speculum examination, the cervix was impossible to visualize, and slight bleeding was observed. On vaginal examination, a solid mass was palpated in the pouch of Douglas, and the external uterine orifice was displaced above the symphysis pubis. Based on these findings, uterine incarceration was suspected. To reduce the incarceration and relieve her symptoms, she was instructed to assume a knee-to-chest position after micturition. However, at 18 weeks and 4 days of gestation, the abdominal pain and genital bleeding persisted and the physical findings remained unchanged. At 18 weeks and 5 days of gestation, magnetic resonance imaging (MRI) was additionally performed to obtain more detailed findings. MRI showed a large fibroid engaged in the pouch of Douglas and a cranioventrally stretched cervix. The uterus was strongly retroverted; therefore the fundus and the posterior wall of uterus were entrapped in the pelvis between the sacral promontory and pubic symphysis (Figure 1). Based on MRI findings, the patient was diagnosed with uterine incarceration and threatened abortion.

After that, she remained in the hospital and continued with the same management; however, the maneuver was unsuccessful. Therefore, manual reduction of the incarceration was planned. However, at 19 weeks and 5 days of gestation, IUFD occurred. Transvaginal and transrectal manual reduction was immediately attempted under general anesthesia to achieve vaginal delivery; however, the attempts were unsuccessful. An expectant management approach was planned, expecting a decrease in the uterine blood flow, leading to reduction in the size of the uterine cavity. We planned to follow up the patient once a week on an outpatient basis by speculum and pelvic examination for less than 4 weeks. Blood tests during the expectant management showed no signs of infection or coagulopathy. The minimum blood fibrinogen level before delivery was 335 mg/dl.

**Figure 2:**
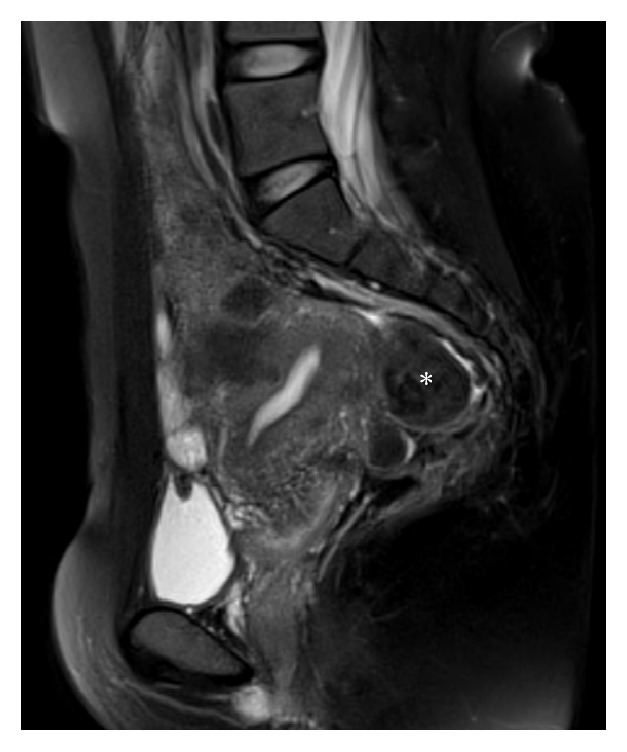
Sagittal T2-weighted MRI after delivery shows a large fibroid (white asterisk) located in fundus.

At 22 weeks and 3 days of gestation (19 days after IUFD), the cervix was visually recognized on speculum examination. On pelvic examination, the fibroid in the pouch of Douglas was still palpated, but the external uterine orifice was palpated in the normal position. At 23 weeks and 5 days of gestation (28 days after IUFD), the incarcerated uterus of the patient resolved spontaneously with reduction of the uterus; subsequently, labor was induced with gemeprost vaginal suppository after mechanical dilatation of the uterine cervix. The macerated fetus was successfully delivered. The patient had a favorable course after delivery and was discharged uneventfully. MRI performed 3 months after delivery showed a large fibroid in the fundus of the uterus (Figure 2). Uterine fibroid may cause recurrence of an incarcerated uterus on the next pregnancy; hence, we performed laparoscopic myomectomy and adhesiolysis of the adhesion between the uterine posterior wall and the rectum.

## 3. Discussion

We reported a case of successful vaginal delivery following IUFD by expectant management in a woman with an incarcerated uterus. Based on the findings of this case and those of previously reported cases, we propose a therapeutic strategy for the management of IUFD in the second or third trimester in women with an incarcerated uterus.

To the best of our knowledge, there are three case reports on IUFD in the second or third trimester in women with an incarcerated uterus. Our case is the fourth one (Table 1). In the first case, the patient was diagnosed with IUFD at 23 weeks of gestation. An attempt to reduce the uterus failed. Subsequently, vaginal delivery was induced despite the presence of her incarcerated uterus, but this attempt also failed. Finally, vaginal delivery was achieved after spontaneous rupture of the membranes [7]. In the second case, the patient was diagnosed with IUFD at 28 weeks of gestation. Several attempts of manual reduction were unsuccessful. Finally, cesarean delivery was performed [7]. In the third case, the patient was diagnosed with IUFD at 21 weeks of gestation. Vaginal delivery was induced despite the presence of her retroverted uterus but was unsuccessful. Subsequently, manual reduction of the uterus was performed, resulting in successful uterine reduction, after which induction of delivery was attempted again; this resulted in a successful vaginal delivery [8]. A case of induced abortion in the second trimester in a woman with an incarcerated uterus has also been reported. Manual reduction was attempted but was unsuccessful; finally, cesarean delivery was performed [9]. However, these reports lack information regarding gestational age at delivery or the time between the diagnosis of IUFD and delivery. These reports have not mentioned the cause of IUFD.

Complications of an incarcerated uterus include miscarriage and IUFD [3, 10, 11]. Although the cause of IUFD is unknown, decreased uterine arterial blood flow by malposition of the uterus may play a role [3, 10]. The reason for fetal demise in the present case is also unclear. However, the reduction of blood flow may be one of the factors associated with IUFD.

The findings of these cases suggest that vaginal delivery may be possible after successful reduction of the incarceration. If the reduction is impossible, expectant management can be an option to enable spontaneous reduction of the incarcerated uterus, so as to achieve vaginal delivery.

There are two advantages of expectant management. First, blood flow to the uterus decreases after IUFD, leading to softening and loosening of fetal tissues and reduction in the placenta size. The reduction in the uterine volume decreases uterine flexion, which may lead to spontaneous resolution of the incarcerated uterus. In addition, amniocentesis, which was not performed in this case, may be effective for reducing the uterine volume. Second, expectant management allows spontaneous onset of labor and subsequent vaginal delivery. It is known that spontaneous labor usually begins within 3 weeks of fetal death in approximately 90% of the cases [12]. If patients go into spontaneous labor during expectant management, they are allowed a trial of vaginal delivery without medical intervention. However, careful monitoring is required when labor begins in women with an incarcerated uterus. If the delivery does not progress as expected, an increased risk of uterine rupture should be considered. Accordingly, cesarean delivery is necessary.

Complications of expectant management include intrauterine infection and coagulation disorder [13–15]. Pritchard reported that coagulation disorder (blood fibrinogen level < 150 mg/dl) did not occur within 5 weeks of IUFD [16]. However, he also reported that coagulation disorder (blood fibrinogen level < 100 mg/dl) was possible to occur after 6 weeks of IUFD [14]. Therefore, one may assume that expectant management for less than 4 weeks can be safely performed with a regular blood test. In the present case, the patient was followed up with blood tests once a week, and there were no signs of infection or coagulopathy during the remaining pregnancy.

No change in the retroflexion of the uterus after 4-week expectant management indicates that the risk of intrapartum uterine rupture still persists; in such cases, cesarean delivery should be considered. The possibility of spontaneous reduction of the uterus decreases as the fetus grows, probably making vaginal delivery difficult. Therefore, the effectiveness of expectant management is to be evaluated separately for cases of IUFD in women in the latter stage of the second trimester and those in the third trimester.

## 4. Conclusion

In summary, in cases of IUFD in women with an incarcerated uterus, vaginal delivery may be possible after successful reduction of the uterus. If the reduction is impossible, expectant management can be an option for reduction of the incarcerated uterus, in order to achieve vaginal delivery. However, careful and individualized management of IUFD is required in women with an incarcerated uterus.

## Figures and Tables

**Table 1:** Summary of cases of IUFD in the second or third trimester in women with an incarcerated uterus.

Number	Author	Year	Age (years)	Gravida/para	GA at IUFD	Result of manual reduction	Delivery method
1	Van Beekhuizen	2003	40	0/0	23	Unsuccessful	Vaginal delivery after spontaneous rupture of the membranes
2	Van Beekhuizen	2003	33	0/0	28	Unsuccessful	Cesarean delivery
3	Matsushita	2014	36	0/0	21	Successful	Vaginal delivery after successful manual reduction
4	Current case	2016	37	0/0	19	Unsuccessful	Vaginal delivery after spontaneous reduction by expectant management

GA, gestational age (weeks); IUFD, intrauterine fetal death.
